# A Supplementary Approach for Effective Anti-Doping Education: A Pilot Study Applying Refutation Texts to Modify Misperception of the Whereabouts System

**DOI:** 10.3390/ijerph19042097

**Published:** 2022-02-13

**Authors:** Zhangyan Deng, Jinyang Guo, Dong Wang, Zuosong Chen

**Affiliations:** 1 School of Education, Shanghai Jiao Tong University, Shanghai 200240, China; zhangyandeng@sjtu.edu.cn (Z.D.); guojinyang@sjtu.edu.cn (J.G.); 2 Department of Physical Education, Shanghai Jiao Tong University, Shanghai 200240, China; tsuwangdong@sjtu.edu.cn; 3 School of Kinesiology and Health, Capital University of Physical Education and Sports, Beijing 100191, China

**Keywords:** refutation texts, misperceptions, doping, whereabouts

## Abstract

Background: Over the past twenty years, a multifaceted anti-doping system was established to detect, deter, and prevent doping among athletes. However, perception of the whereabouts system has been a controversial issue. This pilot study aimed to evaluate the effects of refutation text intervention on the perception of the whereabouts system. Methods: In two studies, we tested whether (1) detailed refutation texts are perceived as more effective than simply refuting with a true or false claim among 132 athletes (47.73% female, mean age = 20.99 ± 2.11), and if (2) refutation text intervention can alter the perception of the whereabouts system among 177 athletes (53.11% female, mean age = 21.17 ± 2.27). Descriptive statistics were calculated, followed by a one-sample T-test, independent T-test, chi-square test, and a repeated-measures analysis of variance. Results: The results demonstrate that five true/false statements were developed as refutation texts, and the mean accuracy of the true/false test is less than the probability of guess (*p* < 0.05, *d* = −0.18). In addition, detailed refutation texts evoked significantly greater perceived effectiveness than the simple refutation texts (*p* < 0.01, *d* = 0.66). Furthermore, the refutation text intervention enhanced the positive perception of the whereabouts system (*p* < 0.01, η^2^ = 0.15). Conclusions: Our findings support the efficacy of refutation texts to improve the misperception of anti-doping regimes among athletes and have implications for future education prevention initiatives.

## 1. Introduction

Over the past twenty years, the prevalence of prohibited substances among elite and sub-elite athletes has been a remaining issue in anti-doping work [1,2]. The use of prohibited substances contributes to the development of numerous physical and mental diseases [3]. In response to these emerging challenges, a multifaceted anti-doping system was established to detect, deter, and prevent doping among athletes [4]. Recently, some researchers reviewed the perceptions of the anti-doping system and found that the perceived effectiveness regarding the whereabouts system has been a controversial issue among athletes [4,5,6].

In 2005, the World Anti-Doping Agency established the online whereabouts system. Elite athletes must report their daily whereabouts for out-of-competition testing at any time [7,8]. On the one hand, athletes affirmed the effectiveness of the whereabouts system in detecting dopers [9,10]. On the other hand, several prominent athletes criticised the effectiveness of the whereabouts system. It has been noted that one-third of elite athletes found filing whereabouts information problematic [11]. In addition, three-quarters of elite Danish athletes consider reporting whereabouts as too time-consuming, and 41% feel a reduced joy of being an elite athlete [10]. Perceived effectiveness plays a crucial role in fostering acceptance and compliance with an organisation and its rules [12]. Thus, there is an urgent need for more research to identify effective intervention strategies to reverse the controversial perception regarding the whereabouts system. What we know about the intervention is primarily based on practical measures. For example, the ADAMS system was updated to make whereabouts management convenient for athletes. To our knowledge, few studies have given sufficient consideration to strengthen the perception of the whereabouts system.

Previous studies have suggested that inducing subjects to consider the opposite might reduce cognitive bias [13,14]. Refutation texts are often presented as effective interventions for changing readers’ naive understandings of topics, with frequent mentions in popular and academic work as promising solutions to scientific [15] and socio-political misconceptions [16]. These texts are defined as those that describe a common misconception, belief, or idea, explicitly refute it, and then offer a satisfactory alternative [17,18]. Recently, Heddy and colleagues [19] found that refutation text intervention elicited a change in attitude toward genetically modified foods. Moreover, Lyu, Fu, and Wang [20] found that refutation text intervention reinforced patients’ trust toward the doctor. These findings provide insight into reducing athletes’ cognitive bias towards the whereabouts system. 

To date, the role of refutation texts in improving perceived effectiveness has not been empirically investigated. Indeed, refutation texts have been used in the anti-doping education programme. For example, WADA added feedback training regarding nutrition supplements into the Athlete Learning Program about Health and Anti-Doping (ALPHA) programme to facilitate learning about the risks of nutritional supplements. However, the refutation texts currently used in anti-doping education programmes still do not provide an explanation of correct answers. This measure is inconsistent with the Knowledge Revisions Components (KReC) framework [21]. Kendeau and O’Brien [22] suggested that providing a clear and coherent explanation is critical in reducing cognitive bias. Thus, the relationship between refutation texts and perceived effectiveness needs further exploration. 

Given the research gap in the field, this pilot study had two main objectives. The first purpose was to examine whether athletes could recognise their knowledge about doping was insufficient after receiving refutation text intervention. The second objective was to explore the effects of refutation text intervention on the perception of the whereabouts system.

## 2. Materials and Methods

### 2.1. Participants

In this study, a cluster sampling method was used to recruit participants. A total of three hundred nine athletes were recruited from the five sports universities in China. There were one hundred thirty-two participants from two sports universities (47.73% female, mean age = 20.99 ± 2.11) who participated in evaluating the refutation texts; one hundred seventy-seven participants from three sports universities participated in the intervention study (53.11% female, mean age = 21.17 ± 2.27). The mean training history of the participants was 7.98 ± 2.36 years. The eligibility criteria for participation were (1) native Chinese speaker; (2) competing in any sport; (3) no self-reported history of any mental disorder; (4) never used the whereabouts system before.

The sample size was calculated using G*Power 3.1 software (Universität Düsseldorf, Düsseldorf, Germany). A minimum of 126 and 128 subjects was necessary for the independent T-test and repeated-measures analysis of variance (RM-ANOVA), assuming a type I error of 5%, statistical power of 80%, a medium effect size, multiple groups, and response variables. 

### 2.2. Procedure and Materials

**Figure 1:**
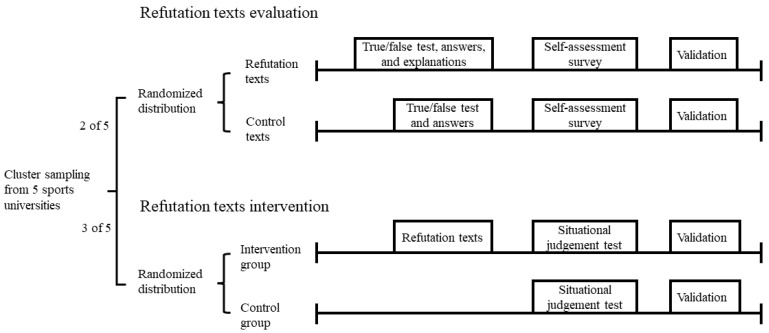
The flow chart of sampling and testing.

The sampling and testing procedure are shown in Figure 1. Participants were surveyed in groups and informed consent was obtained from the sports team leaders and athletes before administration. The questionnaires were completed anonymously in order to protect the participants’ identities. Participants were required to respond honestly and independently. In addition, participants were assured that their responses would be kept strictly confidential and used only for research purposes. It took about 10–15 min for the participants to complete all items, which were collected at the training centre.

During the refutation text evaluation, participants were assigned to receive refutation texts or control texts based on biased coin randomisation. This study organises five true/false statements from WADA Play True Quiz [23,24] and social media rumours about doping (e.g., Therapeutic Use Exemption application), which cover the content of anti-doping tests, nutritional supplements, and therapeutic use exemptions (Table S1). As suggested by Kendeou et al. [22] and Lyu et al. [20], the structure of the refutation texts consists of three elements: (1) questions (call attention to a specific misconception), (2) answers (directly ‘refute’ that misconception), (3) explanations and sources of correct answers (support the refutation with an explanation based on evidence). The refutation texts contained 342 words and were determined to be at a 10th to 12th grade reading level according to Flesch–Kincaid. The control text questions and answers (true or false) were identical to the refutation texts but excluded the explanations and sources of correct answers. After viewing and answering the refutation/control texts, all participants were asked to assess the effectiveness of the texts (‘Through reading and answering these questions, I found that my anti-doping knowledge was not always correct’) on a 5-point Likert scale (1: disagree; 5: agree) and to answer one of five refutation texts again. The participants who mis-answered were excluded from further analysis. A total of 150 questionnaires were distributed to the participants and 132 were included in this analysis. The effective rate of the questionnaire survey was 88%.

The procedures of refutation text intervention consisted of three steps. Step 1, participants were assigned to an intervention or a control group based on biased coin randomisation. The intervention group received the refutation text intervention. The control group received no intervention. By using the control group (which received no intervention), we could be sure that any change that occurred in the intervention condition would be due to the refutation texts. 

Step 2, all participants were asked to complete a situational judgement test. This test was modified [20] to measure the perception of the moral nature of the whereabouts initiative. Since Woolway et al. [5] found that athletes’ perceptions of whereabouts systems are seen as values, fairness, and effectiveness, this study did not directly ask participants to evaluate the effectiveness of whereabouts systems. 

The content of the situational judgement test focused on the statement: ‘submit the whereabouts information by yourself’. Previously, several elite Chinese athletes might have consigned an entourage to submit whereabouts information. However, this initiative is associated with an increased risk of whereabouts failure [25]. Recently, elite Chinese athletes were recommended to submit whereabouts information independently. On the one hand, this initiative decreased the number of whereabouts failures [26]. On the other hand, several athletes found filing whereabouts information problematic, as well as their foreign counterparts [11], and saw this initiative as an attempt to pass the buck. This situation aligns with and further supports the studies describing the misperception of whereabouts policy in Chinese athletes.

The situational judgement test was presented as a dialogue through a series of four pictures. The first picture describes the background, in which the coach informs the protagonist that he will be added to the Registered Testing Pool (RTP) and to submit his whereabouts information soon. The second picture describes the meaning of the whereabouts system. The third picture explains why RTP athletes should submit whereabout information by themselves. The fourth picture displays an alternative option to submit whereabouts information, in which athletes can have their agent or another representative submit their whereabouts information if they wish [27]. In addition, the athlete was reminded that they were ultimately responsible for their whereabouts (e.g., they cannot avoid responsibility by blaming their representative for not updating their whereabouts if they were not at the location specified by them during the 60 min time slot). 

**Figure 2:**
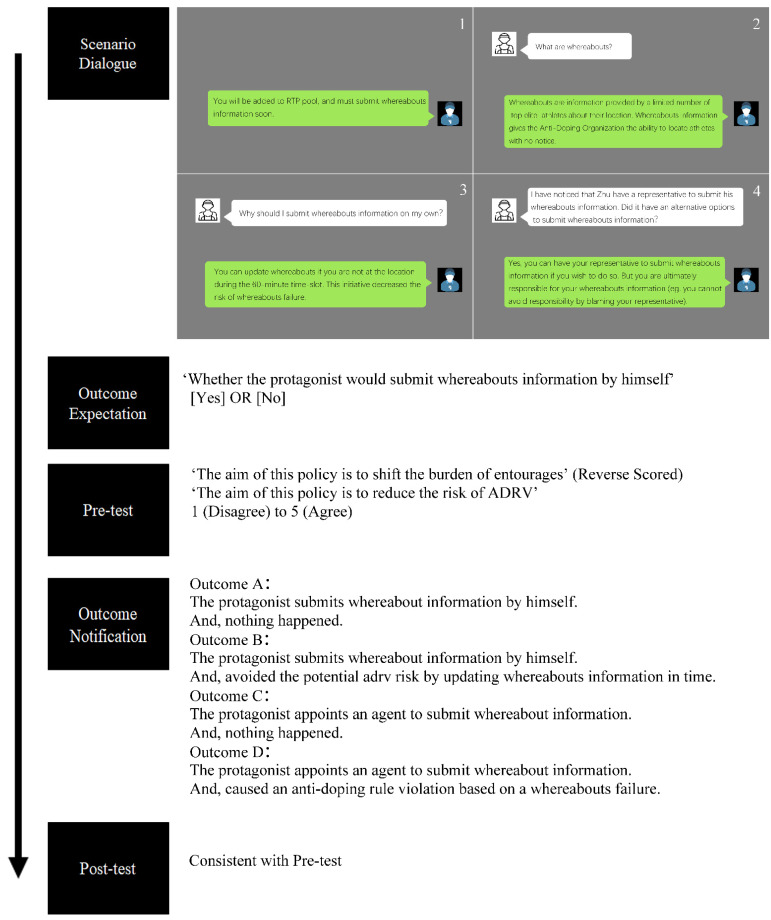
The situational judgement test.

After viewing the dialogue, participants were asked to predict whether the protagonist would submit whereabouts information by himself and to subjectively rate their perception of the whereabouts initiative (Item 1: ‘This policy aims to shift the burden of entourages; Item 2 ‘This policy aims to reduce the risk of Anti-Doping Rule Violation’) on a 5-point Likert scale (1: disagree; 5: agree). Item 1 was reverse-scored so that a high total score indicated a positive perception of the whereabouts policy. Then, the participants were noticed from four resulting conditions. As depicted in Figure 2, the protagonist’s action or inaction could produce either a negative outcome (Anti-Doping Rule Violation, ADRV) or a neutral outcome (nothing happened). Participants were assigned to the four resulting conditions with a block size equivalent using the R package blockrand [28]. Finally, all participants have to re-evaluate their perception of the whereabouts policy.

Step 3, all participants were asked to recognise the family name of the protagonists’ teammate (correct answer: Zhu). Those participants who mis-answered were excluded from further analysis. A total of 200 questionnaires were distributed to the participants and 177 were included in this analysis. The effective rate of the questionnaire survey was 88.5%.

### 2.3. Data Analyses

The statistical analyses were performed using IBM SPSS software for Windows. Assumptions of data normality were examined graphically. One-sample T-tests were performed to determine whether the mean difference in the accuracy of the true/false test was statistically significant from 0.5. An independent T-test was performed to assess any difference in perceived effectiveness between refutation texts and control texts. In addition, chi-square analysis was used to test for behavioural expectation differences between the intervention and control groups. Last, the situational judgement test scores for each outcome (A, B, C, and D) were assessed by a 2*2*2 RM-ANOVA. Greenhouse–Geisser corrections were applied where the assumption of sphericity was violated. The Bonferroni method correction was used to adjust multiple comparisons. Significance levels for all comparisons were set at *p* < 0.05 (two-sided). 

## 3. Results

### 3.1. Refutation Text Evaluation

The demographic characteristics of the participants are shown in Table 1. No significant differences in true/false test scores were observed across gender, competitive level, and athletic event.

**Figure 3:**
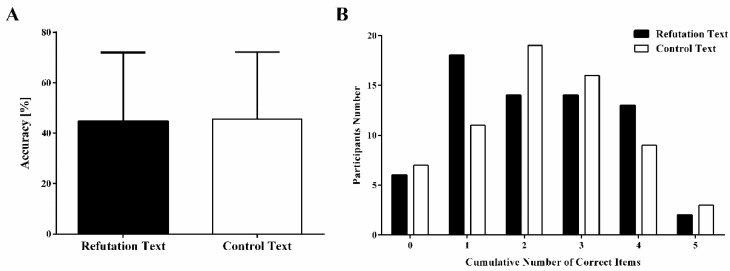
Accuracy (**A**) and the cumulative number of correct items (**B**) of the true/false test.

As depicted in Figure 3, the mean accuracy of the true/false test is significantly lower than the probability of guessing (t_131_ = −2.08, *p* < 0.05, and *d* = −0.18). In total, 96.21% (127/132) of participants could not answer all the questions correctly. In addition, no significant difference in true/false test accuracy was found between the two groups. Moreover, the perceived effectiveness scores for the refutation texts were greater than the control texts (4.14 ± 0.86 vs. 3.46 ± 1.16, t_130_ = 3.79, *p* < 0.01, *d* = 0.66). 

### 3.2. Outcome Expectation

There was no significant difference in behavioural expectation between the two groups (χ^2^ = 3.02, *df = 1*, and *p* > 0.05). In total, 79.07% (68/86) of participants in the intervention group predicted that the protagonist would submit the whereabouts information independently. Comparatively, 67.03% (61/91) of participants in the control group predicted that the protagonist would submit the whereabouts information by himself. 

### 3.3. Situational Judgement Test

**Figure 4:**
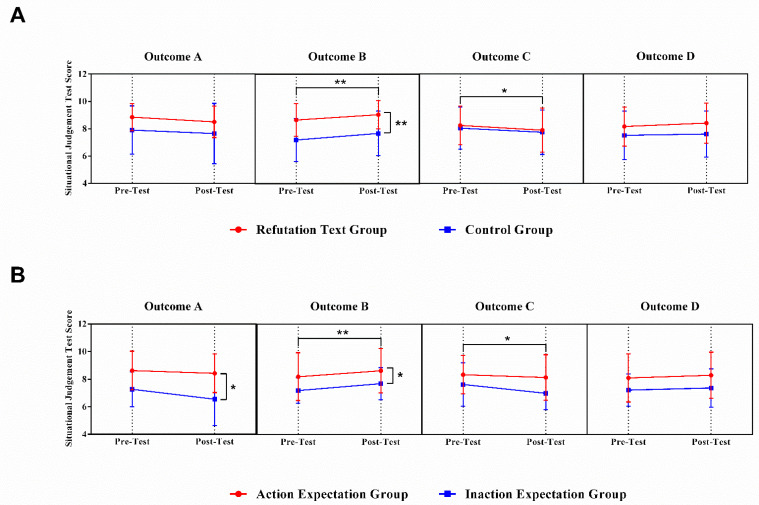
Situational judgement test score for each outcome. (**A**) The difference in situational judgement tests between pre-test and post-test for refutation texts and control group. (**B**) The difference in situational judgement tests between pre-test and post-test for action expectation and inaction expectation group. Data are mean ± SD. * = *p* < 0.05; ** = *p* < 0.01.

As depicted in Figure 4, the results of the RM-ANOVA pertaining to outcome A indicate a significant main effect for outcome expectation (F (1, 42) = 4.96, *p* < 0.05, and η^2^ = 0.11). Compared with the participants who predicted that the protagonist would submit the whereabouts information by entourages, the participants who predicted that the protagonist would submit the whereabouts information on their own reported higher perceived effectiveness regarding whereabouts policy. 

The results of the RM-ANOVA pertaining to outcome B indicate a significant main effect for time (F (1, 40) = 8.47, *p* < 0.01, and η^2^ = 0.18), intervention group (F (1, 40) = 7.28, *p* < 0.01, and η^2^ = 0.15), and outcome expectation (F (1, 40) = 6.62, *p* < 0.05, and η^2^ = 0.14). Firstly, compared with the pre-test score, the participants reported a higher post-test score. In addition, compared with the control group, the intervention group reported higher situational judgement test score. Thirdly, compared with the participants who predicted that the protagonist would submit the whereabouts information by entourages, the participants who predicted that the protagonist would submit the whereabouts information independently reported higher test scores regarding the whereabouts policy. 

The results of the RM-ANOVA pertaining to outcome C indicate a significant main effect for time (F (1, 40) = 4.96, *p* < 0.05, and η^2^ = 0.11). Compared with the pre-test score, the participants reported lower post-test scores. 

Repeated-measures ANOVA indicated no significant main effects or interaction effects on outcome D.

## 4. Discussion

This pilot study aimed to assess the effectiveness of the refutation texts. The results demonstrate that the developed doping-related true/false test could be used as refutation texts. In addition, the refutation text intervention enhanced the positive perception of the whereabouts initiative. 

With regard to the refutation text material evaluation, the mean accuracy of the true/false test is less than the probability of guessing, which confirmed the misconception of doping knowledge for the participants. Our results align with the revisions components framework [22]. This theoretical model suggests that prior misconception is critical in successfully triggering the co-activation and integration of the new ideas. In addition, the refutation texts evoked significantly greater perceived effectiveness than the control texts. This finding is consistent with Ecker et al. [29], who found that detailed refutation is more effective than simply refuting a true or false claim. Similarly, Nyhan and Reifler [30] suggested that detailed refutations are associated with a more sustained reduction in false beliefs. As previously stated, simple refutation texts have been used in anti-doping education initiatives. Taken together, detailed refutation texts might reduce misconceptions about the myth of doping more effectively than simple refutation texts.

Recently, several studies have explored a potential relationship between refutation texts and attitude. Thacker et al. [31] found that refutation texts reinforced approval attitudes toward genetically modified foods. Lyu et al. [20] found that refutation texts enhanced approval perception of medical decision making. It has been reported that misperceptions are associated with negative attitudes [32,33]. In line with previous studies, refutation text intervention enhances the approval perception of the new whereabouts initiative in China. 

With regard to outcome expectation, refutation text intervention increases the likelihood of policy endorsement. Previous research has shown that refutation texts could promote deliberative thinking [20]. Deliberative thinking has been characterised as central to wisdom [34,35]. Grossmann, Brienza, and Bobocel [36] found that wise deliberation led to more moral concerns and sustained cooperation. In addition, recent work has suggested that deliberative thinking is positively associated with a preference for utilitarian decision making [37]. In the perspective of wise deliberation or utilitarian decision making, the benefits (avoiding the risk of ADRV) outweigh the cost (their own time to submit or upgrade whereabouts information) of the whereabouts initiative. Thus, we hypothesised that refutation text intervention might promote wise deliberation or deliberative thinking to accept the whereabouts initiative.

In the current study, we proposed two types of actions that the participants’ prediction could produce, either an expected or unexpected outcome. We observed that the expectation could affect the perception of the whereabouts system under several resulting conditions. With regard to outcomes A and B, the protagonist chooses to follow the whereabouts initiative. Thus, the outcome is expected for participants who choose to predict the protagonist’s action and are more likely to have a positive perception. It is well known that when the outcome of a weather forecast is not consistent with the actual weather, the trust in that forecast is reduced [38,39]. It can thus be suggested that the expected outcome might contribute to a positive perception of the whereabouts initiative. 

After viewing the outcome, the participants were asked to re-evaluate the perception of the whereabouts policy. The results of this module indicate that neutral outcomes enhanced the perception of the whereabouts initiative. In contrast, negative outcomes decreased the perception of the whereabouts initiative. This finding confirms the positive relationship between outcome transparency and legitimacy perceptions [5]. In addition, Engelberg et al. [40] and Hoff [41] disclosed the difficulties in establishing credible deterrents to the athletes who have committed ADRV. Moreover, Murofushi et al. [42] showed that anti-doping education was ineffective in reinforcing participants’ knowledge of the side effects of doping. Thus, developing effective prevention strategies should be considered for the future of anti-doping education.

The International Standard for Education, published in 2021, advocates that an athlete’s first experience with anti-doping should be through education rather than doping control. In this context, future investigations are required to integrate refutation text intervention into anti-doping education programmes. On the one hand, refutation texts may serve as a booster session. MacArthur et al. [43] found that education programmes were more effective with the inclusion of booster sessions. Thus, effective anti-doping education programmes should include booster sessions to help reinforce key messages [44]. On the other hand, refutation texts may serve as a brief priming intervention. Traditional anti-doping education programmes tend to provide knowledge about anti-doping first rather than forming an attitude [45]. However, it has been suggested that a brief priming intervention on self-affirmation might enhance the effectiveness of anti-doping education [46,47]. Since individual attitudes and beliefs mediate the effectiveness of education intervention [48], the anti-doping education programmes should include brief priming intervention to form attitudes, beliefs or perceptions at the initial session.

This pilot study verified the effectiveness of refutation texts to modify misperception regarding the whereabouts system. Our results indicate that refutation text intervention is a feasible strategy for improving the perception of anti-doping legitimacy. Several limitations need to be acknowledged. Firstly, to avoid the knowledge interferences on perception, the modified refutation texts and situational judgement test were used for this pilot study [20]. Indeed, it should be noted that the content of refutation texts (anti-doping tests, nutritional supplements, and therapeutic use exemptions) is indirectly associated with situational judgement tests (whereabouts). However, the current anti-education programme barely discusses different contents in the same module. Taking these changes into consideration, the practical value of the current study is restricted. Thus, future investigations are required to integrate the refutation texts into the current anti-doping education programme. Secondly, the study lacks an active control group. Although, it has been suggested that the effect of refutation text intervention on the conception of education policy is more significant than the other change-promoting instruction, such as explanation-only texts [49,50]. Nevertheless, we do not know the intervention programme’s effect compared with an active control group. Thirdly, the study only examined the acute effects of refutation text intervention. Therefore, it is unclear whether our findings extend to long-term interventions. Future investigations are required to evaluate the long-term impact of the refutation text intervention.

## 5. Conclusions

This pilot study represents the first attempt to modify the misperception of whereabouts system. Some helpful information and new insights in doping research were provided. This pilot study indicates that the developed doping-related true/false test can be used as refutation texts. The refutation text intervention enhances the positive perception of the whereabouts initiative. 

Additionally, we propose that several factors should be considered as modulating factors that, if pronounced, promote the perception of anti-doping measures. In this case, legitimacy perception should be addressed by prediction consistency and outcome transparency. While further improvement can be aimed for in future research, this suggests that even a brief intervention may be worthwhile for helping athletes establish a positive experience with unfamiliar anti-doping measures that could protect them against doping initiation in the future.

## Figures and Tables

**Table 1:** Participants’ characteristics.

	Refutation Text Evaluation		Refutation Text Intervention	
	Refutation Texts *n* = 67	Control Texts *n* = 65	Intervention Group *n* = 86	Control Group *n* = 91
Female %	50.74%	44.62%	51.16%	54.94%
Age	21.02 ± 1.99	20.95 ± 2.23	21.43 ± 2.20	20.93 ± 2.37
Competitive Level	National (*n* = 27) Regional (*n* = 40)	National (*n* = 22) Regional (*n* = 43)	All national	All national
Athletic Event	Team sports (*n* = 24) Individual sports (*n* = 43)	Team sports (*n* = 22) Individual sports (*n* = 43)	Team sports (*n* = 43) Individual sports (*n* = 43)	Team sports (*n* = 42) Individual sports (*n* = 49)

## Data Availability

The data and material are available upon reasonable request.

## Supplementary Materials

The following supporting information can be downloaded at: https://www.mdpi.com/article/10.3390/ijerph19042097/s1, Table S1: Refutation Texts Material.
