# Supplementary material for: A Supplementary Approach for Effective Anti-Doping Education: A Pilot Study Applying Refutation Texts to Modify Misperception of the Whereabouts System
Source: Int J Environ Res Public Health. 2022 Feb 13;19(4):2097. doi: 10.3390/ijerph19042097 (PMC8872570; doi:10.3390/ijerph19042097)
Supplement: Supplementary file 1 [file ijerph-19-02097-s001.zip › ijerph-1538404-supplementary.pdf]

Table S1. Refutation Texts Material.

Item	Answer & Explanation
I can be drug tested during a competition, even if I did not compete.	**Answer: True**<br>Explanation: if you are named as a member of a team, you may be included in the selection for doping control, whether you competed or not [1,2].
If I know a supplement has been through a quality control process, I can be guaranteed that it does not contain any substances on the prohibited list.	**Answer: False**<br>Explanation: There is no way to guarantee that supplements are free of prohibited substances as this industry is not regulated. No organisation can guarantee the safety of any dietary supplement or its content. Eating a balanced diet of natural, whole foods is the best way to improve athletic performance [1,2].
Some athletes are granted therapeutic use exemption (TUE) when they are not needed, allowing them an edge in performance.	**Answer: False**<br>Explanation: In theory, an athlete should get a TUE only after an independent committee – which, according to Wada, should have at least three sports medicine physicians on it – has met and made a recommendation to a sporting body or a national anti-doping organisation. In addition, there is no meaningful association between being granted a TUE and the likelihood of winning a medal [1,2].
A positive test is the only way an athlete can be sanctioned.	**Answer: False**<br>Explanation: The presence of a prohibited substance in an athlete's sample is only 1 of 11 Anti-Doping Rule Violations. Refusing or failing to submit to Sample Collection, Possession of a Prohibited Substance, Complicity, and prohibited Association are some of the other ADRVs that an athlete can be sanctioned for [1,2].
The person who receives my sample at the laboratory knows who I am.	**Answer: False**<br>Explanation: The documentation which accompanies your sample to the laboratory does not reveal your identity. The only information the lab receives is the sport/event/gender/Federation and the date of the test [1,2].

Reference

1. Codella, R.; Glad, B.; Luzi, L.; La Torre, A. An Italian campaign to promote anti-doping culture in high-school students. *Front. Psychol.* **2019**, *10*, 534. https://doi.org/10.3389/fpsyg.2019.00534. eCollection 2019.
2. Hoberman, J. How much do we (really) know about anti-doping education. *Perform. Enhanc. Health* **2013**, *2*, 137–143. https://doi.org/10.1016/j.peh.2014.09.002.
